# Molecular typing and profiling of topoisomerase mutations causing resistance to ciprofloxacin and levofloxacin in *Elizabethkingia* species

**DOI:** 10.7717/peerj.5608

**Published:** 2018-09-12

**Authors:** Ming-Jr Jian, Yun-Hsiang Cheng, Cherng-Lih Perng, Hung-Sheng Shang

**Affiliations:** 1 Graduate Institute of Medical Science, National Defense Medical Center, Taipei, Taiwan; 2 Division of Clinical Pathology, Department of Pathology, Tri-Service General Hospital, National Defense Medical Center, Taipei, Taiwan

**Keywords:** *Elizabethkingia* spp., Fluoroquinolone resistance, gyrA, Molecular typing, High-resolution melting

## Abstract

**Objectives:**

Several *Elizabethkingia* species often exhibit extensive antibiotic resistance, causing infections associated with severe morbidity and high mortality rates worldwide. In this study, we determined fluoroquinolone susceptibility profiles of clinical *Elizabethkingia* spp. isolates and investigated the resistance mechanisms.

**Methods:**

In 2017–2018, 131 *Elizabethkingia* spp. isolates were recovered from specimens collected at tertiary care centers in northern Taiwan. Initial species identification using the Vitek MS system and subsequent verification by 16S rRNA sequencing confirmed the presence of *Elizabethkingia anophelis* (*n* = 111), *E. miricola* (*n* = 11), and *E. meningoseptica* (*n* = 9). Fluoroquinolone susceptibility was determined using the microbroth dilution method, and fluoroquinolone resistance genes were analyzed by sequencing.

**Results:**

Among *Elizabethkingia* spp. isolates, 91% and 77% were resistant to ciprofloxacin and levofloxacin, respectively. The most prevalent alterations were two single mutations in GyrA, Ser83Ile, and Ser83Arg, detected in 76% of the isolates exhibiting fluoroquinolone MIC between 8 and 128 μg/ml. Another GyrA single mutation, Asp87Asn, was identified in two quinolone-resistant *E. miricola* strains. None of the isolates had alterations in GyrB, ParC, or ParE. We developed a high-resolution melting assay for rapid identification of the prevalent *gyrA* gene mutations. The genetic relationship between the isolates was evaluated by random amplified polymorphic DNA PCR that yielded diverse pulsotypes, indicating the absence of any temporal or spatial overlap among the patients during hospitalization.

**Conclusion:**

Our analysis of fluoroquinolone-resistant *Elizabethkingia* spp. isolates provides information for further research on the variations of the resistance mechanism and potential clinical guidance for infection management.

## Introduction

The genus *Elizabethkingia* has been recently revised to include several species based on whole-genome sequencing analysis (Doijad et al., 2016; Nicholson et al., 2017). *Elizabethkingia* species are non-motile, non-fastidious, and glucose-non-fermentative gram-negative bacilli (Janda & Lopez, 2017). Three species, *Elizabethkingia meningoseptica*, *E. miricola*, and *E. anophelis*, are known to cause diseases in humans (Green, Murray & Gea-Banacloche, 2008; Jean et al., 2014; Lau et al., 2016). Recent studies suggest that certain strains causing sporadic cases of meningitis and bacteremia, previously identified as *E. meningoseptica*, belong to *E. anophelis* (Chew et al., 2017; Lin et al., 2017). Several outbreaks of *E. anophelis*-associated infections have been reported, including two outbreaks in the US Midwest in 2016 with 65 confirmed cases (Coyle, 2017; Janda & Lopez, 2017). *Elizabethkingia* species also cause outbreaks in intensive care units as emerging pathogens of nosocomial infections with a high mortality rate and severe morbidity in critically ill patients (Jean et al., 2014; Lau et al., 2016; Opota et al., 2017; Hu et al., 2017). Because of their ability to accumulate different resistance mechanisms and a growing number of more vulnerable hosts, the prevalence of multidrug-resistant *Elizabethkingia* species has increased in the past decades, limiting the options for treatment (Janda & Lopez, 2017; Jean et al., 2014). For instance, resistance to carbapenems is mediated by metallo-β-lactamases (Breurec et al., 2016; Chen et al., 2017; Colapietro et al., 2016). A previous report indicated that fluoroquinolones are suitable for treating *E. meningoseptica* bacteremia (Huang, Lin & Wang, 2018), and empirical evidence indicates that they are effective in treating *E. anophelis* and *E. miricola* infections (Coyle, 2017; Figueroa Castro et al., 2017; Green, Murray & Gea-Banacloche, 2008; Zdziarski et al., 2017). However, a detailed analysis of fluoroquinolone-resistant *Elizabethkingia* spp. infections has not yet been performed.

Fluoroquinolones, such as ciprofloxacin (CIP) or levofloxacin (LVX), have two bacterial drug targets, DNA gyrase and DNA topoisomerase IV (Khodursky, Zechiedrich & Cozzarelli, 1995; Kreuzer & Cozzarelli, 1979). Each enzyme is a heterotetramer, with gyrase composed of two GyrA and two GyrB subunits and topoisomerase IV composed of two ParC and two ParE subunits. Mechanisms of fluoroquinolone resistance include mutational alterations in drug target affinity, increased efflux pump expression, and acquisition of resistance-conferring genes (Hooper & Jacoby, 2016). Single amino acid changes in either gyrase or topoisomerase IV can cause quinolone resistance. In gram-negative bacilli, mutations have been typically localized to the amino-terminal region of the primary target, GyrA (Yoshida et al., 1990), a region conserved among all potential quinolone targets. Mutations in these conserved regions of GyrB, ParC, and ParE are also known to confer fluoroquinolone resistance, like the amino-terminal GyrA region (Heisig, 1996; Yoshida et al., 1991). Accordingly, the genomic DNA regions encoding the conserved protein regions of GyrA, GyrB, ParC, and ParE have been termed quinolone resistance-determining regions (QRDRs).

In this study, we aimed to assess the relationship between the quinolone-resistant phenotype of clinical *Elizabethkingia* spp. isolates in Taiwan and mutations in their DNA gyrase and DNA topoisomerase IV genes.

## Materials and Methods

### Bacterial isolates

In 2017–2018, 131 isolates of *Elizabethkingia* spp. (*E. anophelis*, *n* = 111; *E. meningoseptica*, *n* = 9; *E. miricola*, *n* = 11) were recovered by bacterial culture from respiratory tract, urine, catheter tip, and blood specimens collected at the Tri-Service General Hospital, tertiary care centers in northern Taiwan. The species were initially identified using the Vitek MS system with the IVD 3.0 database (BioMérieux, Marcy-l’Étoile, France). Isolates identified as *Elizabethkingia* species using a previously reported study (Cheng et al., 2018). Briefly, MALDI-TOF spectral analysis software identified significant species-specific peaks to create reference masses for efficient and accurate identification of *Elizabethkingia* spp. All bacterial isolates were kept frozen until used in this study.

### Antimicrobial susceptibility

MIC of CIP and LVX were determined using the broth microdilution method. The susceptibilities were evaluated according to guidelines published by the Clinical and Laboratory Standards Institute (CLSI) including antibiotic-specific breakpoints (CIP: susceptible ≤1 μg/ml, resistant ≥4 μg/ml; LVX: susceptible ≤2 μg/ml, resistant ≥8 μg/ml).

### DNA extraction

Genomic DNA was isolated using a previously reported protocol (Syn & Swarup, 2000). Briefly, cellular lysis is achieved by a combination of EDTA/SDS detergent lysis and brief heat treatment. An additional phenol/chloroform step further deproteinates the preparation yielding DNA of good quality. Using a picodrop spectrophotometer, purified genomic DNA concentrations were determined by measuring the optical density at 260 nm, whereas the purity was estimated by calculating the ratio of the optical densities measured at 260 and 280 nm. DNA samples were stored at −20 °C until PCR was performed.

### Bacteria species identification by 16S rRNA sequencing

The microbial identification accuracy was verified by 16S rRNA sequencing using a pair of specific primers, 27F (5′-AGAGTTTGATCMTGGCTCAG-3′) and 1492R (5′-GGYTACCTTGTTACGACTT-3′), as previously described (Chang et al., 2014). DNA sequencing were compared to reference sequences using the basic local alignment search tool of the National Center for Biotechnology Information database.

### PCR and DNA sequencing of the topoisomerase gene

Isolates were screened for mutations in the *gyrA*, *gyrB*, *parC*, or *parE* genes by PCR using species-specific primers (Table 1). PCR products were sequenced for detection of nucleotide polymorphism. Primers were commercially synthesized by Genomics (New Taipei city, Taiwan). The reaction mixture (50 μl) contained 10 mM Tris–HCl (pH 7.5), 50 mM KCl, 1.5 mM MgCl_2_, 0.2 mM dNTPs, 10 pmol of the forward and reverse primer, 50 ng template DNA, and 0.8 U of Taq DNA polymerase (Applied Biosystems, Foster City, CA, USA). Amplification was carried out in a ProFlex PCR thermal cycler (Applied Biosystems, Foster City, CA, USA) with one initial denaturation step of 2 min at 95 °C; 40 cycles of a denaturing step of 15 s at 94 °C, an annealing step of 1 min at 48–50 °C with corresponding genes, and an extension step of 1 min at 72 °C; and a final elongation step of 5 min at 72 °C. All PCR products were processed for DNA sequencing (Genomics, New Taipei city, Taiwan) with the same PCR primer sets. Sequencing results in candidate genes from each isolate were compared with the respective reference sequences in the GenBank database (NCBI reference sequences: *E. anophelis*, NZ_CP007547.1; *E. meningoseptica*, NZ_CP016376.1; *E. miricola*, NZ_CP023746.1).

**Table 1:** Primer sequences used in this study.

Primer sequences used to amplify *gyrA*, *gyrB*, *parC*, and *parE* genes in *Elizabethkingia* spp.			
Primer name	Sequence (5′→3′)	Annealing temperature (°C)	Product size (bp)
gyrA-E.species-F*	AGC CCG TTG TTT AAA TCC TGA A	50	743
gyrA-E.species-R	CCC TGT TGG GAA GTC TGG TG		
gyrB-E.species-F	GAT AAT TTC CTT CAT AAA GAG CC	48	
gyrB-E.anophelis-R	CAT TGC CAT ACT GAG CTT GT		905
gyrB-E.meningoseptica-R	TCG AAG TGT TTG CTT TGT CA		896
gyrB-E.miricola-R	GCG TTG TCA TAC TGA ACT TG		903
parC-E.species-F**	GCT CAG TAT GGC AAT GCT AAA A	50	785
parC-E.species-R	TTG CTC TTA CCT TAC CGC CG		
parC-E.meningoseptica-F	TGA CCG GAT CAA CCG AAG TC		
parC-E.meningoseptica-R	CAG GTC GCC TGT TGT TTT GG		
parE-E.species-F	GTA TTC AGT TTA AAA GGT AAA CC	48	
parE-E.anophelis-R	GAA TAT ATT GGG CTT CGA CA		694
parE-E.meningoseptica-R	ACT GAA CTT AGT TTG CCA TAA G		657
parE-E.miricola-R	AGA AAT CGA CAT ATT CAG AGG T		683

Primer sequences used for RAPD-PCR.		
Primer name	Sequence	Target
OPA-10	GTG ATC GCA G	*E. anophelis*
OPB-15	GGA GGG TGT T	*E. meningoseptica*
		*E. miricola*

Primer sequences used for fluoroquinolone HRM analysis assays.			
Primer name	Sequence (5′→3′)	Annealing temperature (°C)	Product size (bp)
gyrA-HRM-E.species-F	TGC CAG AAT TGT TGG AGA TG	50	
gyrA-HRM-E.anophelis-R	TAG CGC AGA GAC CAT GAC TG		102
gyrA-HRM-E.meningoseptica-R	GTG CCA TAC GCA CCA TAG CA		83
gyrA-HRM-E.miricola-R	CTG TGC CAT ACG CAC CAT AG		85

**Notes:**

* gyrA-E.species-F and gyrA-E.species-R could amplify all *Elizabethkinga* species *gyrA* gene (including *E. anohpelis*, *E. meningoseptica*, and *E. miricola*).

** parC-E.species-F and parC-E.species-R could amplify both *E. anohpelis* and *E. miricola parC* gene.

### High-resolution melting assay for *gyrA* mutation screening

Three different reverse primers and one common forward primer with homology to the *Elizabethkingia* spp. *gyrA* gene were designed (Table 1). PCR amplification was performed using the KAPA HRM FAST PCR Kit for preparing the following reaction: 20 μl reaction mix containing one μl template DNA (10 ng), eight μl PCR grade nuclease-free H_2_O, 10 μl KAPA HRM FAST Master Mix, two μl 25 mM MgCl_2_, and 0.5 μl of forward/reverse primer mix (10 μM each). The amplification and high-resolution melting (HRM) curve analyses were conducted on a LightCycler 96 instrument (Roche, Mannheim, Germany) using the following cycling conditions: initial activation at 95 °C for 2 min, 40 cycles at 95 °C for 10 s and at 60 °C for 30 s. The post-PCR melting curve was performed using temperatures between 65 and 95 °C in temperature increments of 0.3 °C.

### RAPD-PCR and capillary gel electrophoresis analysis

RAPD-PCR was performed using primers (Table 1) described previously (Hsueh et al., 1996; Chiu et al., 2000). The reaction mixture (25 μl) contained 10 mM Tris–HCl (pH 7.5), 50 mM KCl, 2.5 mM MgCl_2_, 0.2 mM dNTPs, 15 pmol of the RAPD primer, 50 ng genomic DNA, and 0.8 U of DyNAzyme II DNA polymerase (ABI, ThermoFisher Scientific, Foster City, CA, USA). For every sample, each RAPD reaction was performed at least twice for each DNA extract. Amplification was carried out in a ProFlex PCR thermal cycler (Applied Biosystems, Foster City, CA, USA) with one initial denaturation step of 5 min at 95 °C; 40 cycles of a denaturing step of 1 min at 94 °C, an annealing step of 1 min at 36 °C, and an extension step of 2 min at 72 °C, and a final elongation step at 72 °C for 8 min.

After PCR amplification, the products were analyzed on Qsep100 DNA Analyzer (BiOptic, New Taipei City, Taiwan) according to the manufacturer’s instructions. PCR fragments were applied into a miniaturized single-channel capillary cartridge of the Qsep100 DNA-CE with separation buffer. The run was performed using a high-resolution cartridge with a sample injection protocol of eight kV for 10 s and separation at five kV for 300 s. The DNA alignment markers (20 bp, 1.442 ng/μl, and 5,000 bp, 1.852 ng/μl) and the DNA size marker (50–3,000 bp, 10.5 ng/μl) were obtained from BiOptic. Sample peaks were visualized using Q-Analyzer software (BiOptic, New Taipei City, Taiwan).

### Molecular pattern analysis

Isolates were categorized as identical, similar or unrelated according to their PCR banding patterns. The data were analyzed using GelCompar II software (Applied Maths NV, Sint-Martens-Latem, Belgium). Dice similarity coefficients were calculated and clustering was done by unweighted pair group mean association.

### Data analysis

Statistical significance was determined using Student’s *t*-test (GraphPad Prism. GraphPad Software Inc, San Diego, CA). Differences were considered statistically significant when *P* < 0.05.

## Results

### CIP and LVX susceptibility profiles of *Elizabethkingia* spp. isolates and corresponding resistance mutations

**Figure 1:**
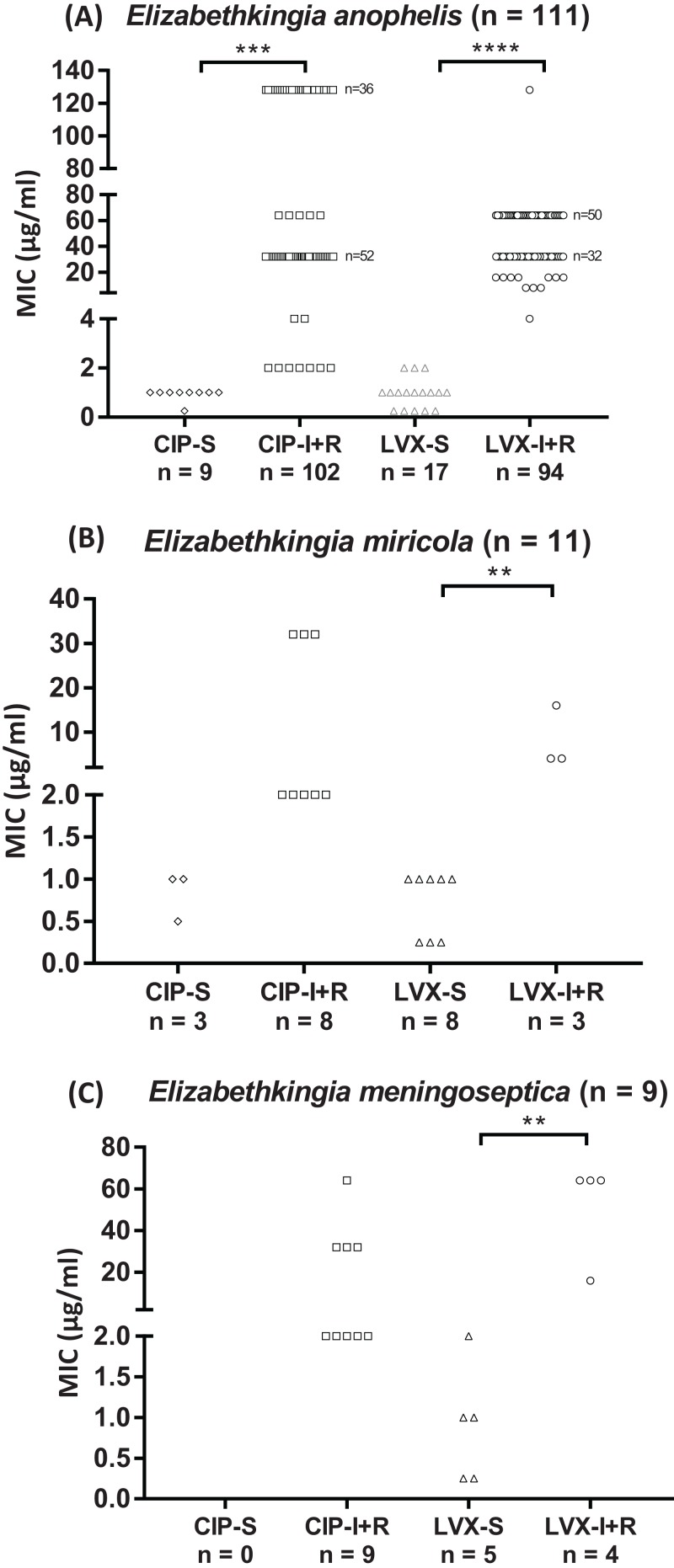
Fluoroquinolone MIC values of *Elizabethkingia* species. (A) *E. anophelis* isolates (*n* = 111). (B) *E. miricola* isolates (*n* = 11). (C) *E. meningoseptica* isolates (*n* = 9). Each symbol (◊, □, ▵, ○) represents one isolate. CIP, ciprofloxacin; LVX, levofloxacin; S, susceptible; I/R, intermediate/resistant Susceptibility (≤ value), intermediate and resistance (≥ value) breakpoints defined by CLSI (2016): one, two and four μg/ml for CIP; two, four and eight μg/ml for LVX. **P < 0.01; ***P < 0.001; ****P < 0.0001.

The 131 *Elizabethkingia* spp. isolates differed in their susceptibility to CIP and LVX (Fig. 1; Table 2); 91% and 77% were resistant to CIP and LVX, respectively. All *E. meningoseptica* isolates were resistant to CIP, whereas 44% were resistant to LVX; 73% of the *E. miricola* isolates were resistant to CIP and 27% were resistant to LVX. Most *E. anophelis* isolates were resistant to CIP and LVX (92% and 85%, respectively).

**Table 2:** Antimicrobial susceptibility of ciprofloxacin/levofloxacin and mutation position detected in the gyrase or topoisomerase IV genes of *Elizabethkingia* species isolates.

Species	Number of isolate	MIC (μg/ml)		Mutation sites			
		CIP*	LVX*	*gyrA*	*gyrB*	*parC*	*parE*
*Elizabethkingia anophelis*	88	32–128	16–128	Ser83Ile	------------ No mutation ---------------		
	6	32	8–64	Ser83Arg	------------ No mutation ---------------		
	17	0.25–2	0.5	-------------- No mutation --------------------			
*Elizabethkingia meningoseptica*	5	32–64	32–64	Ser83Ile	------------ No mutation ---------------		
	4	2	0.25–2	-------------- No mutation --------------------			
*Elizabethkingia miricola*	1	32	16	Ser83Ile	------------ No mutation ---------------		
	2	32	4	Asp87Asn	------------ No mutation ---------------		
	8	0.5–2	0.5	-------------- No mutation --------------------			

**Note:**

* CIP, ciprofloxacin; LVX, levofloxacin. Susceptibility (≤ value) and resistance (≥ value) breakpoints defined by CLSI (2016): one and four μg/ml for ciprofloxacin, two and eight μg/ml for levofloxacin.

A total of 101 (77%) *Elizabethkingia* spp. isolates had single-nucleotide mutations in the QRDR of the *gyrA* gene, whereas no mutations were found in the *gyrB*, *parC*, or *parE* gene of these isolates. In contrast, none of the 30 LVX-susceptible *Elizabethkingia* spp. isolates had mutations in the topoisomerase genes.

Among *E. anophelis* isolates with a *gyrA* gene mutation, 88 (93.6%) had a single-nucleotide mutation resulting in Ser83Ile amino acid substitution, whereas a different nucleotide mutation in six isolates resulted in Ser83Arg substitution. The most common single-nucleotide mutation encoding the Ser83Ile substitution was also found in *E. meningoseptica* and *E. miricola* isolates. Another single-nucleotide mutation in the *gyrA* gene, encoding an Asp87Asn substitution, was found in two *E. miricola* isolates. Our results indicate a strong correlation between the antibiotic susceptibility profiles of the clinical isolates and their mechanisms of fluoroquinolone resistance. The resistance against CIP and LVX in *Elizabethkingia* spp. is mainly mediated by a single-nucleotide mutation in the QRDR of the *gyrA* gene.

The 29 isolates without any mutation in *gyrA*, *gyrB*, *parC*, or *parE* were completely susceptible or had intermediate susceptibility to CIP (MIC, 0.25–2.00 μg/ml) and LVX (MIC, 0.25–2.00 μg/ml), whereas 102 isolates were fully resistant to CIP with a corresponding MIC range of 32–128 μg/ml and an LVX MIC range of 4–128 μg/ml (Table 3).

**Table 3:** Alterations in *gyrA* genes detected by HRM assay and confirmed by DNA sequence analysis in *Elizabethkingia* spp. Isolates.

Species	Number of isolate	Mutation detected by HRM		Confirmation by sequencing
		*gyrA*83	*gyrA*87	*gyrA* gene
*E. anophelis*	94	Mutation	None	Ser83Ile/Ser83Arg
	17	None	None	No mutation
*E. meningoseptica*	5	Mutation	None	Ser83Ile
	4	None	None	No mutation
*E. miricola*	1	Mutation	None	Ser83Ile
	2	None	Mutation	Asp87Asn
	8	None	None	No mutation

### Rapid detection of *gyrA* mutations using the HRM assay

**Figure 2:**
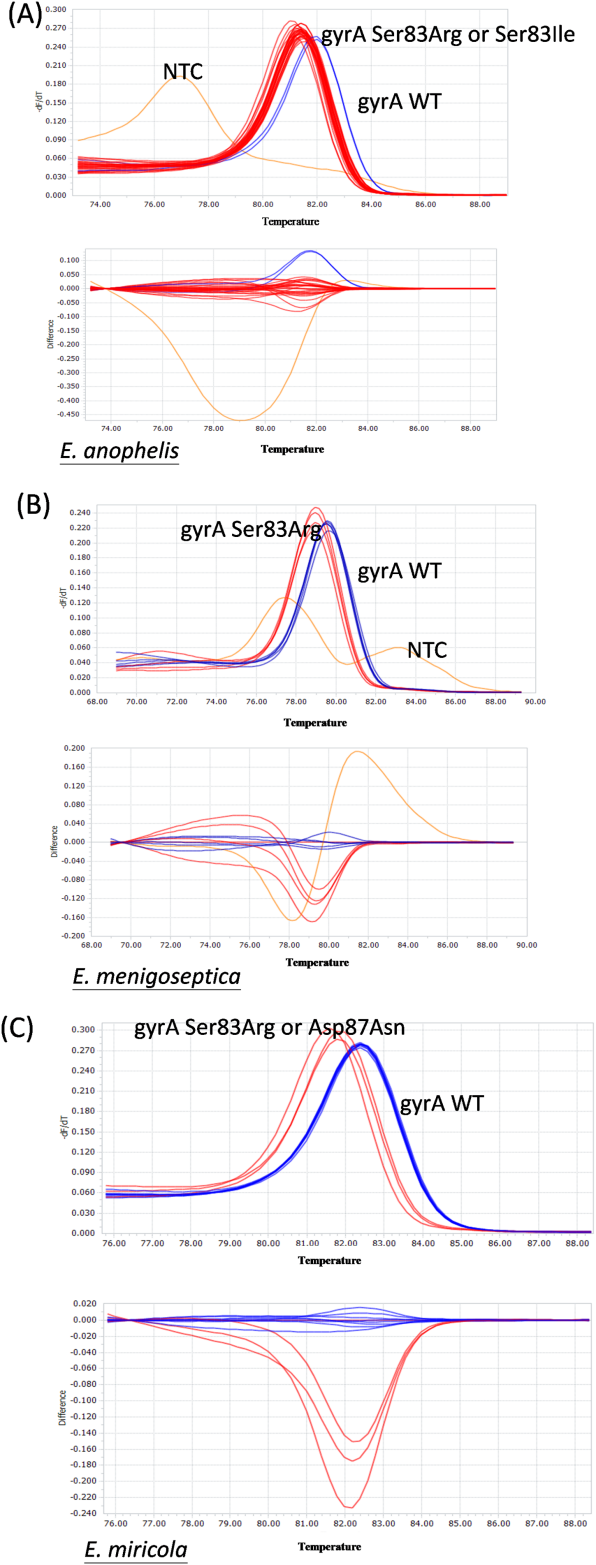
Representative HRM analysis of *gyrA* mutation and wild-type in *Elizabethkingia* species isolates. (A) *gyrA* wild-type (*n* = 2) and *gyrA* mutation (*n* = 28) *Elizabethkingia anophelis* isolates. (B) gyrA wild-type (*n* = 5) and *gyrA* mutation (*n* = 4) *Elizabethkingia meningoseptica* isolates. (C) *gyrA* wild-type (*n* = 8) and *gyrA* mutation (*n* = 3) *Elizabethkingia miricola* isolates. WT, wild-type. Blue lines represent *gyrA* wild-type isolates, red lines and green lines represent *gyrA* mutation isolates, orange lines represent no template control.

The results of the *gyrA* gene sequence analysis of *Elizabethkingia* spp. isolates for the identification of mutations in the QRDR were used to develop an HRM assay that can be used to rapidly scan clinical isolates for typical *gyrA* gene mutations in 131 isolates of *Elizabethkingia* species. The HRM assay successfully detected all *gyrA* mutations in this study, encoding the Ser83Ile, Ser83Arg, and Asp87Asn substitutions (Fig. 2). The HRM assay results for *gyrA* genotyping were in complete agreement with our DNA sequencing results without any exception (Table 3).

### RAPD-PCR typing of *Elizabethkingia* spp. isolates

**Figure 3:**
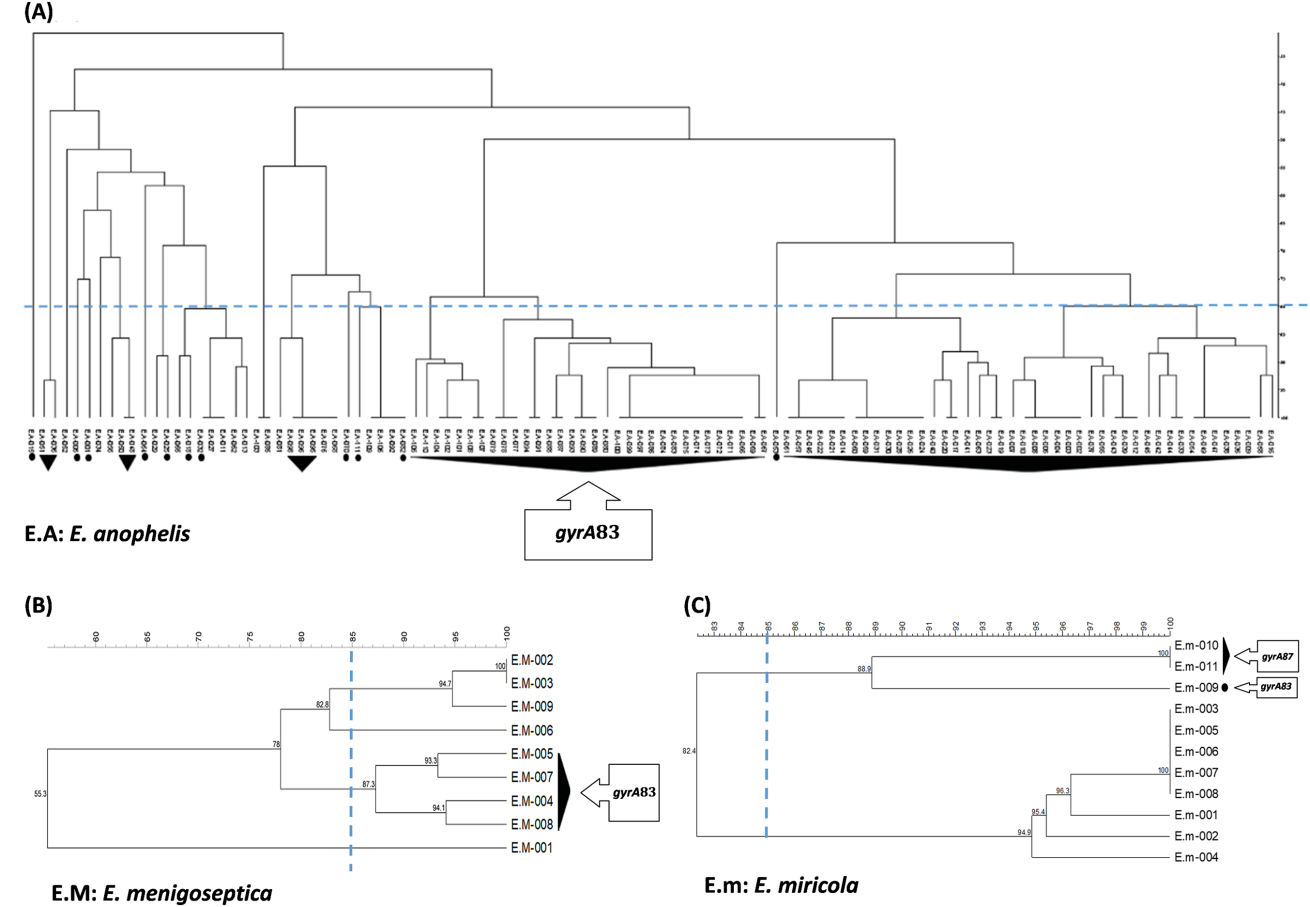
RAPD-PCR dendrogram of the *Elizabethkingia* spp. isolates investigated in this study. (A) Clustering dendrogram of *E. anophelis* isolates (*n* = 111). (B) Clustering dendrogram of *E. meningoseptica* isolates (*n* = 9). (C) Clustering dendrogram of *E. miricola* isolates (*n* = 11). E.A: *E. anophelis* E.M: *E. meningoseptica*; E.m: *E. miricola*. Black triangles represent clusters with multiple isolates possessing the same *gyrA* mutations. Black circles represent monoisolate clusters with *gyrA* mutations. The dashed line represents the similarity level (85%) used in the clusters definition.

The 131 *Elizabethkingia* spp. isolates were clustered into multiple pulsotypes defined by a similarity of ≥85% (Fig. 3). The widespread pulsotype clusters indicated a lack of temporal or spatial overlap among the infected patients during hospitalization. Specifically, pulsotypes of *E. meningoseptica* and *E. miricola* isolates harboring a *gyrA* mutation were found to be distributed among wild type clusters.

## Discussion

*Elizabethkingia* spp. strains represent a group of emerging pathogens, causing infections that are associated with prolonged hospital stays and high mortality rates. In 2015–2016, there was an *E. anophelis* outbreak in Wisconsin, USA, that involved at least 63 patients and 18 deaths (Elbadawi et al., 2016). In addition, another outbreak in Illinois, USA, involving 10 cases with six deaths has also been reported in 2014–2016 (Navon et al., 2016). Globally, there are numerous sporadic *E. meningoseptica* nosocomial infection clusters and *E. miricola* infection case reports in medical centers including in Taiwan. Thus, pathogenic *Elizabethkingia* spp. strains appear to be opportunistic infectious agents associated with high mortality rates.

Quinolones underwent decades of development since the discovery of nalidixic acid in 1962, and quinolone resistance has also existed for decades. Recent studies described LVX-resistant *E. meningoseptica* bacteremia that is associated with an increase in mortality and prolonged hospital stays (Huang et al., 2017; Huang, Lin & Wang, 2018). Appropriate antibiotic use and an effective treatment regime are very important in fighting *Elizabethkingia* spp. infections. Using the broth microdilution method for MIC testing, we found differences in susceptibility to CIP and LVX among the *Elizabethkingia* spp. isolates. The discrepancy might be due to the different CLSI breakpoints, four μg/ml for CIP and eight μg/ml for LVX. Previously reported susceptibility profiles of *E. anophelis* isolates, including from the outbreak in Wisconsin in 2016, indicated that most isolates were susceptible to quinolones (Lau et al., 2016; Perrin et al., 2017). In sharp contrast, among our 111 *E. anophelis* isolates, only nine and 17 were found to be susceptible to CIP and LVX, respectively.

The genetic determinants of quinolone resistance have never been studied in *Elizabethkingia* spp. Our results revealed that certain single-nucleotide substitutions in *gyrA* conferred resistance to CIP and LVX in *Elizabethkingia* spp. The Ser83Ile substitution in GyrA protein was caused by the most prevalent mutation among all isolates, followed by the Ser83Arg or Asp87Asn amino acid substitutions caused by single-nucleotide mutations in *E. anophelis* or *E. miricola*. To our knowledge, this is the first report discussing genetic quinolone resistance determinants in *Elizabethkingia* spp.

Bacterial isolates carrying single alterations in QRDRs of DNA gyrase and topoisomerase IV typically exhibit reduced susceptibility to fluoroquinolones, which is considered as the first step in the development of full resistance (Hooper & Jacoby, 2017). The genetic basis for fluoroquinolone resistance appears to be additive, different combinations of distinct resistance mechanisms may result in different MIC (Conley et al., 2018). Other resistant mechanisms such as plasmid-mediated quinolone resistance might also be involved in the quinolone resistance mechanism (Yugendran & Harish, 2016). In our study, resistance to CIP and LVX was associated with single-nucleotide mutations in the QRDR of the *gyrA* gene in all *Elizabethkingia* spp. isolates causing low-level to high-level fluoroquinolone resistance. The level of fluoroquinolone resistance did not correlate with the type of mutation found in the *gyrA* gene. Other mechanisms typically implicated in fluoroquinolone resistance might be responsible for the differences in CIP and LVX MIC observed among the isolates. Changes in permeability and increased efflux pump activity along with plasmid-encoded resistance determinants cannot be excluded.

In this study, we also established a novel rapid HRM assay for detecting *gyrA* mutations in *Elizabethkingia* spp. The HRM results were in complete agreement with the DNA sequencing results, indicating that we developed a potentially useful adjunct test for the rapid detection of CIP and LVX resistance in *Elizabethkingia* spp.

## Conclusions

Our findings demonstrated that the quinolone resistance in *Elizabethkingia* spp. is associated with mutations in the QRDR of the *gyrA* gene. However, the level of resistance to quinolones of *Elizabethkingia* spp. isolates could not be predicted based on the mutations identified in the *gyrA* gene. This study provided information for further research on the variations of the fluoroquinolone resistance mechanism and potential clinical guidance for infection management.

## Supplemental Information

10.7717/peerj.5608/supp-1 Supplemental Information 1 Raw data of our clinical isolates. All *elizabethkingia* species collected in our study, with MIC values against ciprofloxacin and levofloxacin. We also calculated antimicrobial susceptibility percentage with susceptible/intermediate/resistant in all isolates.

## Additional Information and Declarations

### DNA Deposition

The following information was supplied regarding the deposition of DNA sequences: Our sequences were aligned with the reference sequences via GenBank accession numbers described as follows: *E. anophelis*, NZ_CP007547.1; https://www.ncbi.nlm.nih.gov/nuccore/NZ_CP007547 E. meningoseptica, NZ_CP016376.1; https://www.ncbi.nlm.nih.gov/nuccore/1153881081 E. miricola, NZ_CP023746.1; https://www.ncbi.nlm.nih.gov/nuccore/NZ_CP023746.1

### Data Availability

The following information was supplied regarding data availability: The raw data are provided in the Supplemental Files.
